# Why Do We Still Need a Stable Long Lifetime Deep Blue OLED Emitter?

**DOI:** 10.1021/acsami.1c09189

**Published:** 2021-07-07

**Authors:** Andrew Monkman

**Affiliations:** OEM Research Group, Dept. of Physics, Durham University, Durham, DH1 2UE, U.K.

**Keywords:** OLEDs, hyperfluorescence, hyperphosphorescence, DABNA, organic displays, FRET, Dexter transfer

## Abstract

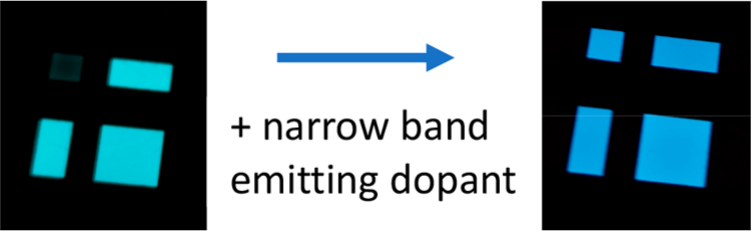

The need for a high efficiency deep blue organic emitter with narrow emission line width has never been so great. This is driven by the need to simplify the complex OLED stack for displays to enable larger substrate sizes to be used and greatly increase production yields. Here, the merits of using the hyperfluorescence scheme based around new multiresonance boron nitrogen molecules typified by DABNA type emitters are discussed and key requirements for suitable sensitizer hosts described, especially the photophysical requirements for optimal performance.

The group currently focus on triplet harvesting using the TADF mechanisms, developing key photophysical models that describe reverse intersystem crossing in a range of different triplet harvesting systems. Prof Monkman has published more than 460 papers with a current H-index of 81.

Current OLED display architectures have diverged into two main technology streams, dependent on production yield. Rather reminiscent of the VHS Betamax video format battle in the 1980s. Samsung pioneered an ambitious display architecture, using individual red, green, and blue sub pixels, Figure 1, left. This design works very well on smaller scale fabrication lines, and is ideal for mobile phone and tablet displays in which they dominate the OLED segment of the market. However, because of the limitations of the fine metal mask technology used, going to much larger sizes of panel becomes problematic, both because of pixel registration due to differential thermal expansion of the huge area 10 μm thick masks and the rapid clogging of the small apertures in the mask. LG on the other hand adopted a simpler approach, using a homogeneous white OLED (effectively unpatterned) as a back light source with printed subtractive color filters on top to define the red, green, blue, and white, 4 subpixel panels, Figure 1, right. This gives much higher fabrication yields for large-area panels, but the displays have restricted color gamut and can suffer differential color aging, particularly in the blue. LG’s technology can be readily scaled to Gen 10.5 glass and beyond and so this technology dominates the OLED TV panel market. However, developing a stable, long lifetime deep blue OLED emitter that harvests 100% triplets will cause a disruptive change to this situation. Figure 2 shows what is considered to be the optimal solution for all OLED panels. Here, the panel consists of one homogeneous blue OLED, that is, a “*blue back plane*” concept, preferable with a single emission layer but it could be a tandem or trilayer architecture depending on achievable IQE and application. Red and green sub pixels are defined by additive down conversion fluorescent filters (potentially using quantum dot, “QD”, fluorescent materials), replacing wasteful subtractive absorptive color filters. This architecture has all the advantageous of the simpler LG type architecture but will give a greater color gamut (BT.2020 and beyond) required for hi-definition 4K and 8K displays but avoids problems associated with differential color aging. This simpler architecture will give easier fabrication, increasing the production yield and so reduce cost. It is also more suited for top emission architectures. Current blue OLED emitters, however, cannot achieve the require performance levels to realize this panel architecture. Phosphorescent iridium(III)-based emitters can yield 100% IQE and around 20–25% EQE, being used commercially for all red and green pixels, but in the blue, they generally suffer from suboptimal IQEs, have difficulty in attaining the required CIE color point, and remain fundamentally unstable having short lifetimes.^1^ This is in part an intrinsic problem as in the deep blue the Ir metal centered d–d* transition is excited which leads to nonradiative decay and photodegradation of the complex.^2^ Blue thermally activated delay fluorescence (TADF) emitters have been realized,^3^ as has 100% IQE and importantly through spontaneous molecular orientation^4^ and by the use of very low refractive index materials throughout the OLED stack, EQEs of 37% have been demonstrated.^5^ But device lifetimes remain poor, well below industrial requirements, realistically a T95 of 5000 h. As yet we simply do not understand TADF well enough as an emission mechanism to propose a viable solution. We do know though that the triplet harvesting rate, the reverse intersystem crossing rate, *k*_RISC_, is slow, at best achieving around 1 × 10^6^ s^–1^ in the solid state when calculated using kinetic modeling^6^ that accounts for nonradiative quenching decay, leading to poor device performance at higher brightness levels, that is, poor efficiency roll-off.

**Figure 1:**
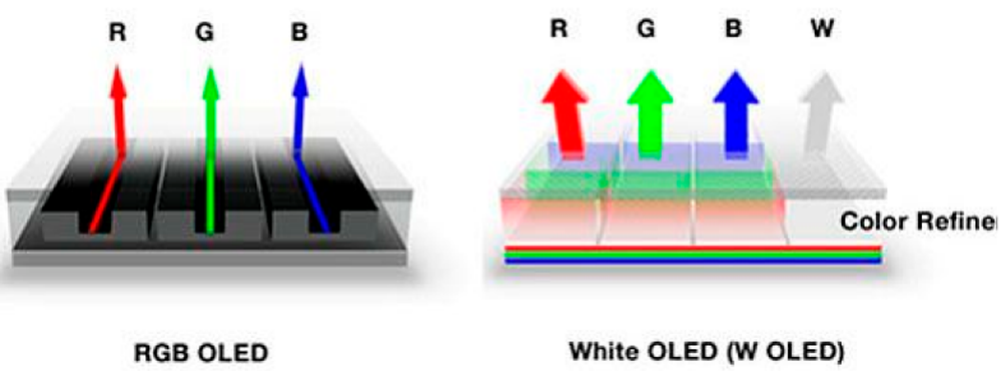
Schematic of (left) Samsung’s RGB OLED structure and (right) LG’s WRGB, white OLED structure.

**Figure 2:**
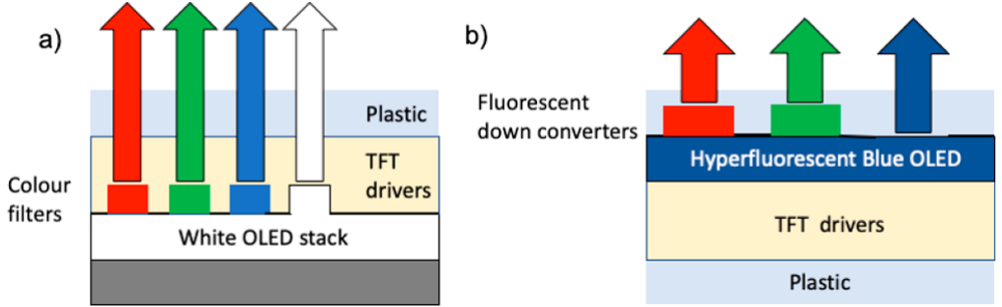
Schematic of (a) WRGB with subtractive color filters, white back plane OLED compared to (b) the more simple blue back plane OLED architecture.

Both Ir and especially TADF emitters also suffer greatly with another major problem, very broad emission spectra, far from giving saturated color. This is also a critically important factor because near saturated colors are required to give the largest overall color gamut for a display. In both cases, subtractive filtering has to be used to achieve saturated colors, reducing EQE from 25% to ∼7–10%. To solve this very difficult problem, sensitized luminescence, either hyperphosphorescence or hyperfluorescence (see Figure 3) has been proposed, where a narrow line width fluorescent emitter is added at low concentration as a codopant within the emissive layer of the device and excited via Förster resonance energy transfer (FRET) from either a phosphorescent^7^ or TADF^8^ triplet harvesting sensitizer. However, for deep blue, this requires a TADF emitter (or Ir emitter) of very high energy to enable the required deep blue emission from the fluorophore because of the FRET energy step down. To achieve the BT.2020 color space standard for example, the blue emission must have CIE coordinates of *x* = 0.145, *y* = 0.046, which is very challenging.^9^ This introduces two further major problems that also need to be overcome: those related to triplet exciton management by (1) preventing the (high) energy triplet states of the sensitizer molecules transferring onto the (lower energy) fluorophore molecules by Dexter transfer before triplet conversion occurs, especially for the TADF sensitizers were both intersystem crossing (ISC) and reverse ISC are orders of magnitude slower than ISC on a typical Ir phosphor and (2) managing direct charge recombination occurring on the fluorophore emitter. Both lead to triplet states accumulating on the hyperfluorescent dopant, thereby causing large efficiency loss, degradation and so short device lifetimes.^8^ Dexter transfer can be reduced using low concentrations of both sensitizer and fluorophore in a host material (typically an ambipolar charge transport material) but direct charge recombination is more difficult to control. Achieving long device lifetimes is always a problem and so aspects of pixel architecture need to be exploited to decrease the stress on the emitter to help as much as possible.

**Figure 3:**
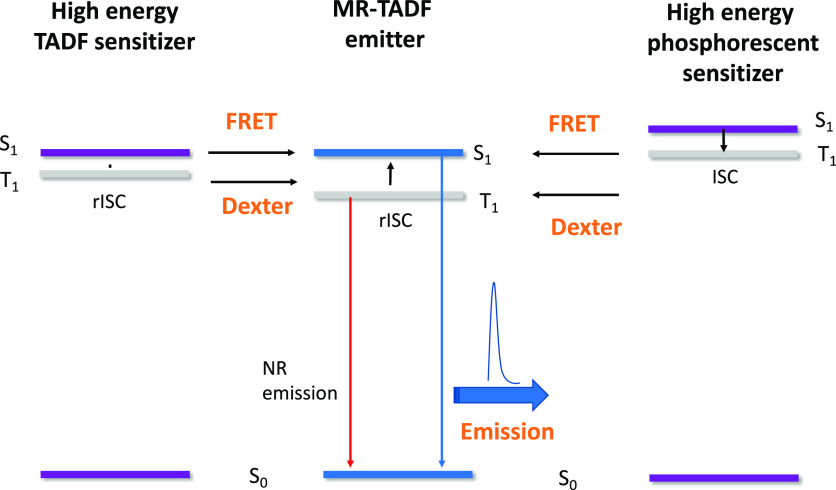
Schematic diagram depicting the various optical decay channels after excitation of the sensitizer, either through optical excitation or charge recombination. Förster resonant energy transfer of either radiative singlet or triplet states (i.e., emissive phosphorescent states) populates the emitter singlet state, Dexter transfer of triplet (and singlet) energy via two electron transfer) populates the emitter triplet states. Reverse intersystem crossing, rISC thermally activates triplet states to convert into the singlet states.

Narrow line width high efficiency fluorophores for sensitized luminescence are now available though, using “multiresonance TADF” DABNA type boron nitrogen molecules, Figure 4.^10,11^ DABNA molecules are very efficient emitters, PLQY > 90%, with full width at half-maximum emission line widths intrinsically as low as 14 nm, and very importantly, very small Stokes shifts. They also triplet harvest via an upper triplet state reverse intersystem crossing (rISC) mechanism,.^12,13^ Thus, triplets transferred accidentally onto the DABNA or formed by direct charge recombination on the fluorophore can be dealt with fairly quickly. This reduces the probability for charge-carrier triplet annihilation on the fluorophore, a potential degradation mechanism. This form of rISC is slow, however, leading to distinct roll-off of efficiency at high brightness, above typically 1000 nits. For a 120 Hz refresh rate display, transient pixel brightness can reach up to 5000 nits which invariable will cause problems, for any triplet harvesting systems with >1 to 2 μs delayed fluorescence or phosphorescent lifetimes. Thus, using the fast and highly efficient fluorescent decay channel of the DABNA in the hyperfluorescence scenario provides the optimum performance and robustness against efficiency roll-off.^14^

**Figure 4:**
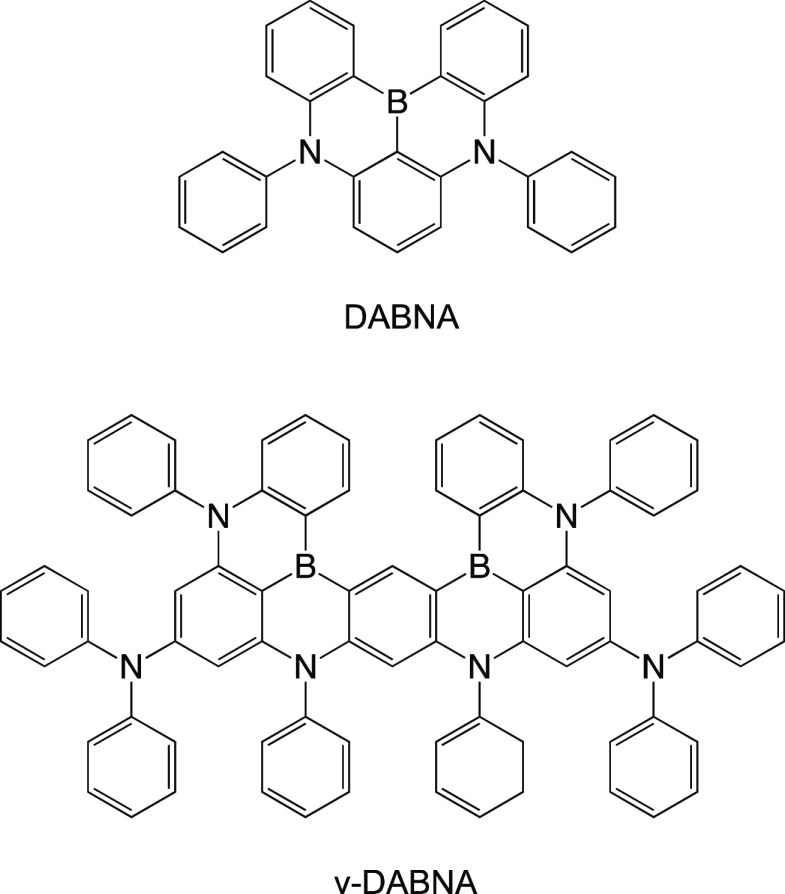
Structure of DABNA and ν-DABNA narrow MR-TADF emitters.

DABNA molecules are rigid planar molecules with high shape anisotropy that readily self-orient during vacuum deposition, greatly enhancing light outcoupling, which is another reason for their extremely high performance as hyperfluorescent fluorophores.^11^ Further, with the orientation coming from the fluorophore, it is independent of the sensitizer host system, making it far easier to optimize each component within the whole emitter layer. This planarity does though introduce problems with dimerization, which limits doping levels that can be used.^12^ Adding bulky side groups is an obvious route to prevent this but will introduce new vibrational modes that broaden the emission spectrum because of strong electron-vibronic coupling, so this will take thought and care to achieve effectively.

To achieve CIE *y* = 0.046 values, the fluorophore needs to emit close to 460 nm, here the very small Stokes shift of DABNA molecules greatly helps us, meaning the TADF triplet harvesting systems needs to emit around 440–450 nm to pump the DABNA via FRET, Figure 3, not any higher in energy. For TADF emitters this could be critical. As FRET drives an optical transition via dipole–dipole coupling it only requires the 0–0 transition (electronic root) of the sensitizer to overlap with the 0–0 absorption of the fluorophore. It is, thus, possible to overlap only the leading blue edge of the sensitizer emission spectrum with the red edge of the sharp DABNA absorption band, Figure 5a, to achieve >95% FRET efficiency at a FRET radii >3 nm, so approximately at concentrations of 0.5–1 wt % of the fluorophore, Figure 5b. In this way, the sensitizer only needs to be a little more blue than the (DABNA) fluorophore given a very small Stokes shift. This will help greatly to achieve usable lifetimes from the sensitizer molecules and gives a strong guide to future sensitizer design. For a phosphorescent sensitizer, the phosphorescence needs to be at about 440–450 nm for good FRET to the fluorophore; note Forster transfer only requires an allowed emission transition dipole moment, which can couple to the absorption transition dipole moment of the accepting fluorophore, but this can mean that the singlet state of the Ir complex ligand is significantly higher than this which could lead to accelerated d–d* Ir excitation. This needs more work to fully understand the photophysics involved and ramifications on stability.

**Figure 5:**
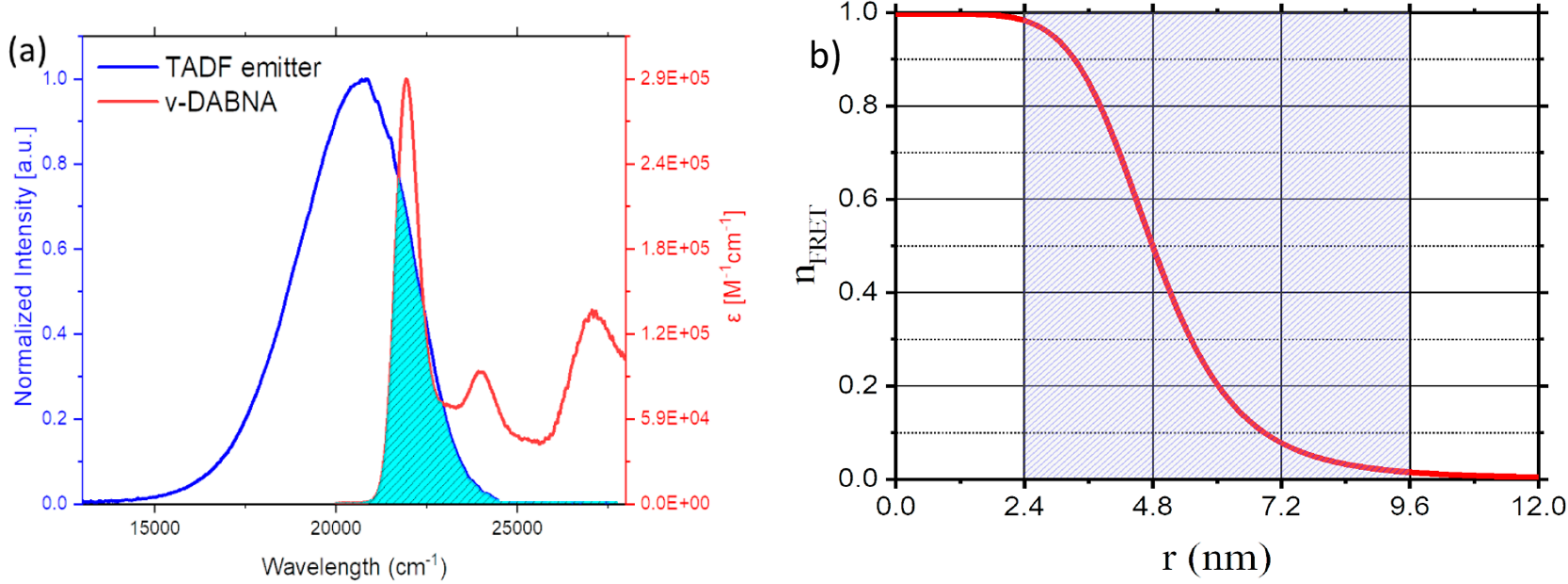
Förster radius analysis. (a) Calculated overlap between normalized sensitizer emission spectra and extinction coefficient of the DABNA fluorophore. (b) FRET efficiency (*n*_FRET_) as a function of average sensitizer-fluorophore separation distance (*r*).

There seems to be contrary evidence that sensitizer-fluorophore FRET can speed up the TADF rISC step by driving the “recycling” process between singlet and triplet charge transfer states (^1^CT–^3^CT) faster into the singlet state.^15^ Limited experimental evidence suggests that the TADF retains the same microsecond triplet residence time, that is, feeding singlets to the fluorophore at the same rate as the DF without a sensitizer.^16^ This could be because the rate limiting step in rISC is the vibronically coupled spin orbit coupling mechanism that mediates the rISC, involving very weakly radiative triplet states that do not spectrally overlap with the fluorophore absorption.^17^ This can leave the TADF molecule open to charge-carrier triplet annihilation and degradation. More work is required to fully understand these complex FRET-TADF mechanisms. For an Ir phosphor sensitizer, ISC is orders of magnitude faster than FRET so the energy transfer rate can be much faster than the intrinsic microsecond phosphorescence radiative lifetime without affecting the triplet harvesting ISC step which might be very important in achieving 440–450 nm emission while avoiding the metal d–d* transition and its associated degradation when excitation is via charge recombination.^18^ This is another open question requiring much more work to fully characterize. However, it does not prevent Dexter transfer of the triplet state from the phosphor sensitizer to the emitter if they are nearest neighbors. Thus, we still have the problem of potential triplet population of the emitter here as well.

The pit falls of Dexter transfer are always discussed alongside hyperfluorescence. If the sensitizer is in close proximity to the fluorophore (within 1 nm or so, i.e. nearest neighbor) then triplet energy transfer via the Dexter mechanism will out compete FRET, causing triplet excitation of the fluorophore which is totally undesirable. This becomes a major problem at both high brightness and high fluorophore doping levels. Shielding of the fluorophore with multiple bulky side groups has been proposed to prevent this but it often causes detrimental red shifting of the emission. Another problem is that the Dexter processes cannot be characterized through optical measurements as FRET totally outcompetes the slow ISC in TADF materials leaving negligible triplet population, however this is not a problem for hyperphosphorescence were ISC is orders of magnitude faster than in TADF molecules.^16^ The DABNA fluorophores with their intrinsic TADF solve this problem rather neatly. However, we now know that the lowest triplet state of highly efficient blue emitting ν-DABNA^11^ is 70 meV below the lowest singlet state and the first excited triplet state is resonant with it,^12,13^ but triplet transfer from the sensitizer must be extremely low, even though the (upper) triplets must align very well given the impressive 41% EQE values at 470 nm peak wavelength reported,^14^ even accounting for enhanced out-coupling effects. This could indicate that triplet harvesting on the ν-DABNA is critically important in this context. But, we need to develop new experimental techniques to study these processes in the TADF hyperfluorescence context.^16^

So, yes there is a great need for new deep blue OLED emitter systems that can be used to realize the blue back plane architecture. Hyperfluorescence has many plus points in its favor, giving the required narrow emission line width and high EQEs with potential device stability, using DABNA type fluorophores. This does though push the problems back onto the triplet harvesting sensitizer system to be used. As discussed above, current triplet harvesting methods still have major problems to be overcome, mostly linked to photostability. One interesting alternate avenue could be exciplex TADF sensitizers.^19^ In this case, the electron and hole (before recombination) reside on separate molecules which could offer increased stability.^20^ Moreover, as we have shown by controlling the donor and acceptor molecule separation with a third spacer molecule, emission can be tuned to the blue, having more local character so faster luminescence decay.^21^ The third molecule can then be used to further optimize the properties of the host systems such as charge mobility etc. This method also benefits from the fact that final emission anisotropy is governed by the DABNA fluorophore, not the uncontrolled excimers so that enhanced outcoupling can still be readily achieved. Again, there is much to be explored here. Overall, hyperfluorescence is tantalizingly close to enabling the blue back plane panel concept; the technology is actively being pursued by Kyulux, Inc., who are making great strides in improving performance and pushing toward its commercialization, Figure 6. We just need to improve the sensitizer molecules or systems to give enough operational lifetime and develop our understanding of the many photophysical processes at play to be able to fully realize the potential of hyperfluorescence OLEDs and the blue back plane OLED architecture.

**Figure 6:**
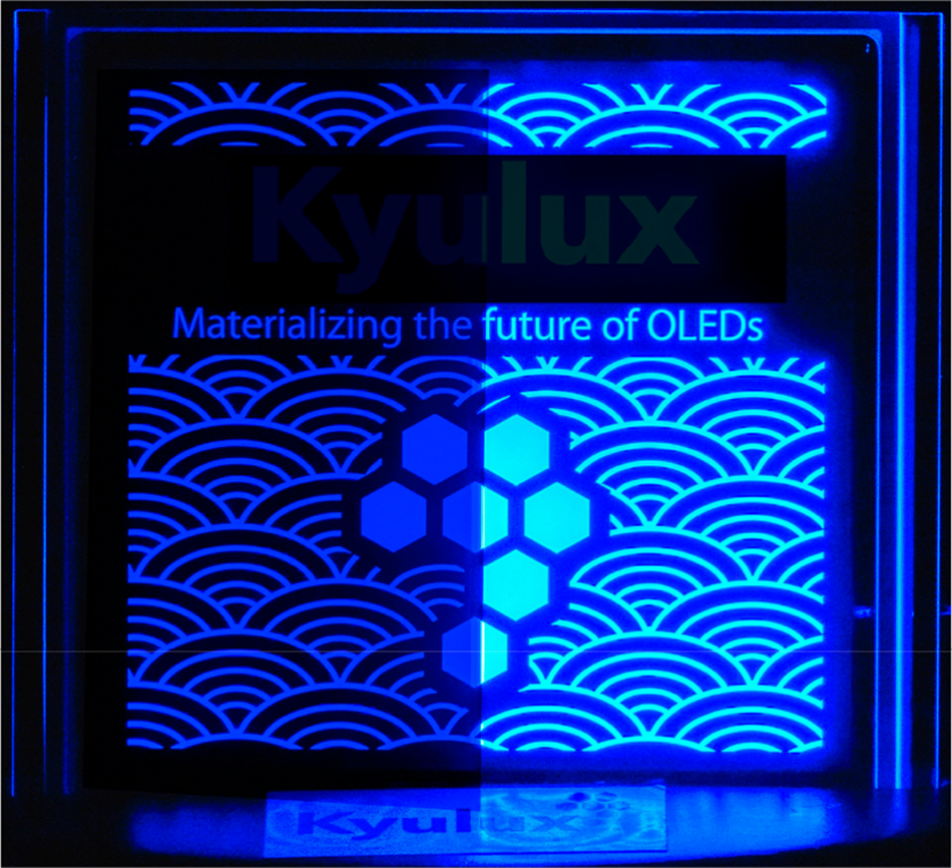
Demonstration of blue hyperfluorescence OLED compared to the sensitizing host on its own. Courtesy of Kyulux Inc.
